# THE MAGIC OF MUSIC FOR IMPROVING PSYCHOLOGICAL HEALTH FOR THOSE WITH DEMENTIA AND THEIR CAREGIVERS

**DOI:** 10.1093/geroni/igz038.2760

**Published:** 2019-11-08

**Authors:** Stuart MacDonald, Debra J Sheets, Andre P Smith, Mary Kennedy, Sandra Hundza

**Affiliations:** University of Victoria, Victoria, British Columbia, Canada

## Abstract

Arts-based interventions for person’s with dementia and their caregivers represent an inexpensive, non-invasive, and non-pharmacological intervention with the potential to improve psychological function as well as reduce healthcare costs. The paper presents an overview of Voices in Motion (ViM), and the impact of this social-cognitive intervention on changes in psychological function for those with dementia and their caregivers (current n=26 dyads). Choir rehearsals were held on a weekly basis, and included a social discussion component. A range of outcomes (neuropsychological and physiological function, neural activation) were assessed using an intensive repeated measures design that facilitates both between- and within-person analyses, including nuanced evaluation of whether psychological function improves post intervention relative to an individual’s personal average, yielding a conservative within-person test of the benefits of intervention. Discussion focuses on the promise of such interventions for mitigating dementia symptoms and facilitating the psychological health of caregivers.

